# Subthreshold Depressive Symptoms have a Negative Impact on Cognitive Functioning in Middle-Aged and Older Males

**DOI:** 10.3389/fpsyg.2013.00309

**Published:** 2013-05-31

**Authors:** Erlend J. Brevik, Rune A. Eikeland, Astri J. Lundervold

**Affiliations:** ^1^Department of Biological and Medical Psychology, University of Bergen, Bergen, Norway; ^2^K.G. Jebsen Centre for Research on Neuropsychiatric Disorders, Bergen, Norway; ^3^Kavli Research Center for Aging and Dementia, Haraldsplass Deaconess Hospital, Bergen, Norway

**Keywords:** aging, depression, subthreshold depressive symptoms, cognition, sex-differences, fluid intelligence, crystallized intelligence, neuropsychology

## Abstract

**Introduction:** Cognitive aging is associated with a decline on measures of fluid intelligence (gF), whereas crystallized intelligence (gC) tends to remain stable. In the present study we asked if depressive symptoms might contribute to explain the decline on gF in a sample of healthy middle-aged and older adults. **Method:** The Norwegian sample included 83 females and 42 males (*M* = 60, SD = 7.9 years). gF was calculated from factor-analysis, including tests of matrix reasoning (WASI), memory function (CVLT-II), processing speed and executive function (CDT; CWIT). gC was derived from a Vocabulary subtest (WASI). Depressive symptoms were assessed by self-reports on Beck’s Depression Index (BDI) and ranged from 0 to 21 (*M* = 6, SD = 4.5). **Results:** Increased age was correlated with a decline on gF (*r* = −0.436, *p*  < 0.001), but not gC (*r*=−0.103, *p* = ns.). The BDI score in the whole sample was correlated with gF (*r* = −0.313, *p* < 0.001). A more detailed analysis showed that the BDI score correlated with measures of both gF and gC in males. The correlations were non-significant for females on all measures, with the exception of a measure of processing speed/executive function. A regression analysis including age and sex in the first step, showed that symptoms of depression significantly contributed to explain decline on gF, *F*(3, 124) = 16.653, *p* < 0.001, *R*? = 0.292, Δ*R*? = 0.054. **Discussion:** The results showed that symptoms of depression were negatively correlated with cognitive functioning in males even when the symptom-level was below clinical threshold. This indicates that minimal symptoms of depression in older men are clinically relevant to address.

## Introduction

Aging is associated with cognitive decline (Salthouse, 2004, 2009). Cross-sectional studies have demonstrated that such a decline begins when an individual is in his or her thirties (Tucker-Drob and Salthouse, 2008). However, longitudinal studies have revealed a more differentiated picture, with large individual differences in cognitive function across all age groups, and no necessary intra-individual cognitive decline (Schaie, 2005; Nyberg et al., 2012). As the population across the World is getting older, knowledge concerning healthy aging and how to retain high cognitive function is increasingly important (Park and Reuter-Lorenz, 2009; WHO, 2011).

Although most elderly people feel that their cognitive function has declined with age, the impact of age on cognitive function is quite differentiated (Park and Reuter-Lorenz, 2009; Nyberg et al., 2012). Fluid intelligence (gF), which represents the capacity to think logically and solve novel problems independent of previously acquired knowledge, tends to show an age-related decline and to be heavily influenced by brain-pathology (Salthouse, 2012). Crystallized intelligence (gC) represents accumulated learning experiences and world knowledge, and is more resistant to change (Lezak et al., 2004; Park and Reuter-Lorenz, 2009), thus providing valuable information about previous functioning. Assessment of these two aspects of cognitive function is therefore valuable in studies of cognitive aging.

Several factors are known to contribute to performance on tests of cognitive functions. At a group level there are significant sex-differences, both regarding cognitive performance (Steinmayr et al., 2010) and how the brain supports cognitive functions (Haier et al., 2005; Burgaleta et al., 2012). Women tend to surpass men in verbal ability tests (Delis and Kramer, 2000) and tests measuring processing speed (Delis et al., 2001), whereas men tend to surpass women in spatial orienting tasks (Wechsler, 1999). The amount and direction of sex-differences in cognition vary somewhat between studies, indicating that the overall sex-differences are small (Steinmayr et al., 2010; Burgaleta et al., 2012). Second, severe symptoms of depression are associated with cognitive dysfunction (Austin et al., 2001; Alexopoulos, 2005; Hammar and Årdal, 2012). The relation is expected to be of great importance in older-age, because it is a period of life when both mood-related (Alexopoulos, 2005) and cognitive problems (Herrmann et al., 2007) are frequently reported. A study by Bush et al. (2001) showed that one should be aware of even mild symptoms of depression, far below the threshold traditionally needed to get medical attention. They showed that both psychiatric morbidity and mortality was increased at very low levels of depressive symptoms (Beck’s Depression scale = 4–9) in a group of patients who had survived an acute myocardial infarction.

Sex counts, both regarding prevalence and expression of depressive symptoms (Piccinelli and Wilkinson, 2000). Females tend to report higher levels of depressive symptoms on self-report measures than males (Tousignant et al., 1987). This shows that reports of depressive symptoms are more prevalent in female than male populations, but it may also reflect that the same level of expressed symptoms has a different meaning and significance for males than for females (Rabbitt et al., 1995). Little is known about the impact of sex on the relation between depressive symptoms and cognitive aging (Gale et al., 2012). In one of the few studies of this relation, Ng et al. (2009) found that depressive symptoms had an impact on the Mini-Mental State Exam score (Folstein et al., 1975) in a sample of older Chinese adults, and that this relation was only significant for males.

This motivated the current study to investigate the impact of mild symptoms of depression on cognitive measures of gC and gF in a group of healthy middle-aged and older male and female adults. From earlier studies we expected that:

(1) Measures of gF- but not gC will be affected by age.
(2) Depressive symptoms will correlate negatively with measures of gF-, but not with gC.
(3) Depressive symptoms will add to the predictive value of age and sex in explaining differences in gF.

## Materials and Methods

### Participants

Healthy individuals were invited through advertisements to take part in the first wave of a longitudinal study on cognitive aging. All participants were interviewed before inclusion, and participants were excluded if they reported present or previous neurological or psychiatric disorders, a history of substance abuse, or other significant medical conditions. Participants were examined according to an extensive neuropsychological test-protocol, including tests used to extract measures of crystallized and gF. The neuropsychological test results were reviewed by experienced neuropsychologists, ensuring that the participants were not suffering from dementia or mild cognitive impairment (MCI) (Petersen et al., 2001). Of the initial participants in the dataset *n* = 163, 11 were excluded because of missing data on the measure of full-scale IQ, or an IQ level below 80 [i.e., 3 SDs below the sample mean of 116.2 (SD = 11.1)]. Another 27 participants were excluded because they lacked a full test-protocol, to ensure the same number of participants on all measures. Of the remaining 125 participants, 83 were females. The age of the participants ranged from 46 to 79 years. All participants had completed obligatory schooling of at least 7 years (see Table 1 for a summary of the sample demographics). The Regional Committee for Medical and Health Research Ethics of Southern Norway approved this first wave of the study, and the participants constitute a subsample of the Norwegian Cognitive NeuroGenetics study (Espeseth et al., 2012).

**Table 1:** **Sample characteristics**.

	*M* (SD)	Females	Males
*n*	125	83	42
Age	60.0 (7.9)	59.4 (7.7)	61.1 (8.4)
Education (years)	14.1 (3.2)	14.2 (3.2)	13.9 (3.4)
BDI score	6.0 (4.5)	5.7 (4.5)	6.5 (4.5)
IQ	116.2 (11.1)	115.8 (11.0)	116.9 (11.4)

### Neuropsychological assessment

The selection of neuropsychological tests used to define gF and gC was inspired by the GWAS study of Davies et al. (2011).

*Fluid intelligence (gF)* was measured by inclusion of the following tests:

(a) The Matrix Reasoning (MR) subtest from Wechsler Abbreviated Scale of Intelligence (WASI) (Wechsler, 1999), a non-verbal abstract problem-solving test where the participants were presented incomplete patterns and asked to complete them by selecting one of five alternative response options. Number of correctly completed patterns was the outcome measure;
(b) A Cued letter Discrimination Task (CDT) (Espeseth et al., 2006), an experimental reaction-time test, where the participants were presented for valid cues predicting the subsequent target location (50% of the trials), invalid cues (16.7%), neutral cues (16.7%), or no cue (16.7%). The participants were told to make rapid and correct responses. Alertness was obtained by asking the participants to categorize stimulus as a vowel or consonant. The overall mean RT across all CDT conditions was used as the outcome measure;
(c) California Verbal Learning Test (CVLT), second edition (Delis and Kramer, 2000), a memory test where a list of 16 words was read to the participants five times (List A). The task was to recall as many words as possible after each presentation. Immediately after the fifth trial, the participants were read a new list (List B) and asked to recall it. Then, the participants were asked to recall the words from List A and then according to given categories (cues). After 30 min, the participants were asked to recall List A words with and without cues. Finally, they performed a recognition test. The present study included three CVLT measures: the number of hits across the five list A learning trials (1–5), and the short- and long-delayed free recall conditions;
(d) The Color-Word Interference Test (CWIT) (Delis et al., 2001), a test including four conditions where the participants: (i) named a set of color patches (red, blue, and green), (ii) read color words, (iii) named ink colors of incongruent color words, and (iv) switched between naming incongruent colors as in the previous condition and just read the words when presented inside a box. The outcome measure was time to finish each of these four conditions.

*Crystallized intelligence (gC)* was measured by performance on the Vocabulary subtest from WASI (Wechsler, 1999). The participants were given a target word and asked to define it. They obtained a score according to the number of definitions scored as complete. The test measures factual knowledge, and the verbal ability to comprehend and express oneself.

*Depressive symptoms* were measured by the score on the Beck’s Depression Inventory-II (BDI) (Beck et al., 1996). The total BDI score ranges from 0 to 63 points, with higher scores indicating increased severity. According to the manual, a score of BDI ≤ 13 indicates no or minimal depression, scores ranging from 14 to 19 indicate mild depression, from 20 to 28 moderate depression, and ≥29 a severe depression.

### Statistics

Raw scores from the neuropsychological tests were z-standardized to obtain the same unit of measurement across all variables. The standardized scores of CDT and CWIT were inverted to obtain the same order as the other three test-scores, with higher scores indicating better cognitive functioning. A hierarchical principal component analysis (PCA) was performed to obtain a gF factor score. First, two separate first-order PCAs were run on the three CVLT scores and the four inverted CWIT scores, respectively. The first unrotated CVLT- and CWIT factor scores were included in further analyses. A second-order PCA was run on the CVLT and CWIT factor scores, the z-standardized MR score, and the z-standardized and inverted CDT RT score, and the first unrotated principal component was used to represent gF. Bartlett’s test of sphericity was highly significant (on both the first and second order PCAs) (Bartlett, 1954), indicating that the data were suitable for hierarchical PCA. The z-standardized raw score from the WASI Vocabulary test was used to represent gC.

Pearson’s correlation analysis was used to investigate relations between age, depression and the neuropsychological measures. A Bonferroni correction for the correlation analyses according to the formula *p* = 0.05/39 = 0.0013 has been run. To compare the sexes, an independent samples *t*-test for the equality of means was run. Levene’s test for the equality of variances made it possible to assume equal variance. Analysis of variance (ANOVA) was used to control for main and interaction effects. All tests were two-tailed.

The predictive value of age, sex, and depressive symptoms in explaining gF was investigated by running a linear regression analysis using the General Linear Model (GLM) package in SPSS, including age and sex as independent variables in the first model, and then adding BDI scores as an independent variable in the second model. gF and its four components were used as dependent variables in separate analyses.

## Results

**Figure 1:**
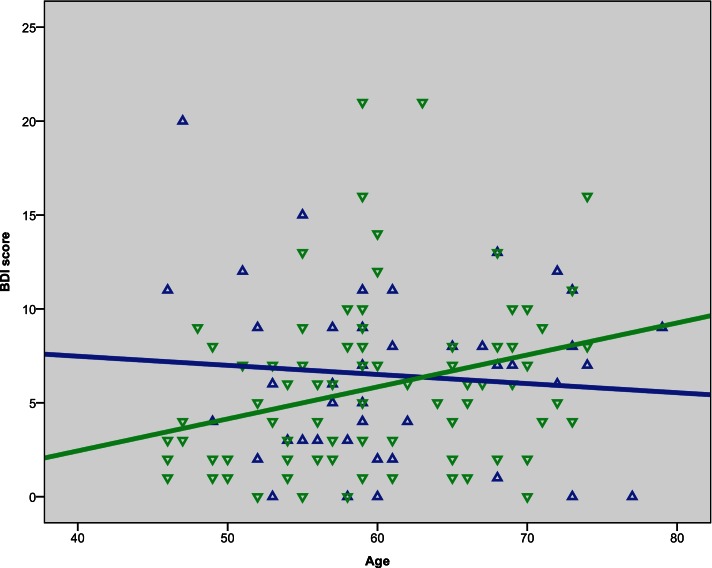
**Scatterplots of age with BDI score**. Green represents female, blue represents male.

The females in the sample were slightly younger and more educated than the males, but the differences were statistically non-significant (Table 1). Symptoms of depression, assessed by the BDI, ranged from 0 to 21. The scores were somewhat higher in males than females, but at a statistically non-significant level (Figure 1). A univariate ANOVA with BDI score as dependent variable showed that the interaction between sex and age was statistically non-significant, *F*(21, 72) = 1.494, *p* = 0.107. Both females and males showed a mean total IQ score in the higher range of the normal distribution.

Performance on the neuropsychological tests used to derive gF and gC is reported in Table 2, with mean scores on each of the tests, their relations to sex, age and depression, and factor loadings from the PCA. In deriving the first order factors in constructing the gF, the Kaiser–Meyer–Olkin (KMO) measure of sampling adequacy was 0.768 for the CVLT factor and 0.681 for the CWIT factor (Kaiser, 1974). The KMO on the second-order PCA leading to the gF factor was 0.592, indicating that the data were suitable for PCA. All test-scores correlated significantly with age, except for the Vocabulary raw score. The females performed significantly better than the males on all subtests of the CVLT, and on the first two conditions of the CWIT.

**Table 2:** **Cognitive tasks used to derive gC and gF**.

					First order PCA		Second order PCA

Cognitive task	*M* (SD)	Sex (*t*)	Age (*r*)	BDI score (*r*)	A	B	C
Vocabulary raw score	65.46 (7.26)	0.12	−0.10	−0.10	–	–	–
Matrix reasoning raw score	24.80 (4.83)	−0.33	−0.18*	−0.19*	–	–	0.56
CDT overall reaction time	679.84 (111.11)	0.73	−0.22*	−0.26**	–	–	0.63
CVLT-II immediate recall (trail 1–5)	51.87 (10.27)	5.06***	−0.29**	0.04	0.94	–	0.65
CVLT-II short delay free recall	11.15 (3.03)	4.77***	−0.28**	0.06	0.94	–	–
CVLT-II long delay free recall	12.02 (3.03)	4.32***	−0.29**	0.01	0.94	–	–
CWIT condition 1-color	29.84 (5.47)	2.35*	−0.26**	−0.20*	–	0.84	0.73
CWIT condition 2-word	20.93 (3.16)	2.97**	−0.23*	−0.24**	–	0.73	–
CWIT condition 3-color/word inhibition	56.55 (13.77)	1.28	−0.42***	−0.26**	–	0.84	–
CWIT condition 4-word inhibition/switching	63.13 (15.01)	0.23	−0.32***	−0.29**	–	0.67	–
% Explained by first component	–	–	–	–	88.04	60.08	41.76

*Correlation is significant at the 0.05 level (2-tailed).

**Correlation is significant at the 0.01 level (2-tailed).

***Correlation is significant at the 0.001 level (2-tailed).A, PCA factor loadings for CVLT; B, PCA factor loadings for CWIT; C, PCA factor loadings for gF.

**Figure 2:**
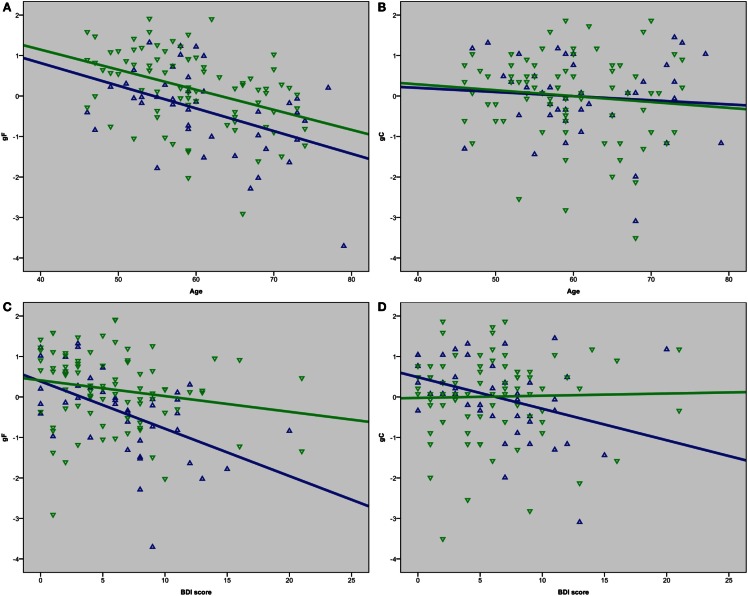
**Scatterplots of (A) age and gF, (B) age and gC, (C) BDI score and gF, (D) BDI score and gC**. Green represents female, blue represents male.

A univariate ANOVA including gF as the dependent variable and age and sex as fixed factors showed significant main effects, *F*(30, 72) = 1.891, *p* = 0.015 for age, and *F*(1, 72) = 11.326, *p* = 0.001 for sex, but no sex–age interaction, *F*(21, 72) = 0.758, *p* = 0.758. Independent *t*-tests between females and males showed statistically significant lower results in males than females on the gF, *t*(123) = −3.0, *p* = 0.003, *d* = −0.54, the CVLT factor score, *t*(123) = −5.1, *p*  < 0.001, *d* = −0.92, and the CWIT factor score, *t*(123) = −2.2, *p* = 0.027, *d* = −0.40 (Table 2). Bivariate correlation analyses showed that age was significantly negatively correlated with gF in both males (*p* = 0.003) and females (*p* < 0.001) (Table 3; Figure 2A). In females, the age-related decline was evident on the CDT score (*p* = 0.020) and the CWIT factor score (*p*  < 0.001), in males on the CVLT factor score (*p*  < 0.001). The correlations with age were non-significant for both sexes regarding gC (Table 3; Figure 2B).

**Table 3:** **Correlation table**.

	BDI score	MR	CDT	CVLT^a^	CWIT^a^	gC	gF
**OVERALL**							
Age	0.162	−0.183*	−0.218*	−0.304***	−0.397***	−0.103	−0.436***
BDI score	–	−0.185*	−0.262**	−0.036	−0.315***	−0.102	−0.313***
**FEMALES**							
Age	0.289**	−0.138	−0.255*	−0.141	−0.459***	−0.110	−0.409***
BDI score	–	−0.121	−0.115	0.044	−0.257*	0.024	−0.189
**MALES**							
Age	−0.089	−0.276	−0.126	−0.519***	−0.270	−0.088	−0.454**
BDI score	–	−0.318*	−0.583***	−0.090	−0.397**	−0.365*	−0.514***

*Correlation is significant at the 0.05 level (2-tailed).

**Correlation is significant at the 0.01 level (2-tailed).

***Correlation is significant at the 0.001 level (2-tailed).

^a^First order factor scores, i.e., higher score = better performance.

Bivariate correlation analyses showed that the BDI score was negatively correlated with gF (*p* < 0.001) and three of its components; MR (*p* = 0.039), CDT (*p* = 0.003), and the CWIT factor (*p* < 0.001), but not gC (*p* = 0.258) (Table 3). Separate analyses within the sex-groups showed that the BDI-age correlation was statistically significant in females (*p* < 0.001), but not in males (*p* = 0.225) (Table 3; Figure 1). Significant negative correlations between BDI score and gF (*p* < 0.001) and gC (*p* = 0.017) were found in males, but not in females (*p* = 0.088 and *p* = 0.829, respectively) (Table 3; Figures 2C,D). The CWIT factor score correlated significantly with the BDI score in both females (*p* = 0.019) and males (*p* = 0.009), while males also showed significant correlations between BDI and both the MR (*p* = 0.040) and the CDT score (*p* < 0.001).

As age is known to influence gF, and sex has been shown to have a substantial effect as well, these two variables were entered in the first model of a hierarchical regression analysis, with BDI score added as a variable in the second model. Overall, the BDI score explained 9.8% of the variance of gF. When age and sex were accounted for, the BDI still explained 5.4% unique variance (Table 4). Separate analyses for the subcomponents of gF showed that the unique variance explained by the BDI score, after accounting for age and sex, was significant in two of the components, 5.2% for the CDT, *R*^2^ = 0.101, Δ*R*^2^ = 0.052, *F*(1, 121) = 4.527, *p* = 0.009, and 6.0% for the CWIT factor, *R*^2^ = 0.242, Δ*R*^2^ = 0.060, *F*(1, 121) = 10.020, *p* = 0.002.

**Table 4:** **Predictive value of BDI score on gF**.

Step	Predictor variable	Δ*F*	*R*^2^	Δ*R*^2^	ß	*p*
	**OVERALL**
1	Age	19.065	0.238	–	−0.413	0.001
	Sex	–	–	–	0.220	0.007
2	BDI score	9.249	0.292	0.054	−0.236	0.003
	**FEMALES**
1	Age	16.228	0.167	–	−0.409	0.001
2	BDI score	0.522	0.172	0.005	−0.077	0.472
	**MALES**
1	Age	10.376	0.206	–	−0.454	0.003
2	BDI score	25.059	0.517	0.311	−0.560	0.001

## Discussion

The present study showed that increased age was associated with lower scores on gF but not gC for both sexes. Furthermore, higher symptoms load of depression was associated with lower gF scores. A more detailed analysis showed that this association was only statistically significant for males, with the same scores on BDI having little influence on cognitive functioning among the female participants. Symptoms of depression showed a negative correlation with gC in males, but not in females. And finally, depression added a unique explanation of the decline on gF, even when age and sex were accounted for. However, separate analyses for the two sex-groups revealed that depression only contributed to the prediction of gF score in males.

The association between age and the included measures of cognitive functioning was expected from earlier cross-sectional studies (e.g., Tucker-Drob and Salthouse, 2008; Nyberg et al., 2012). Age and depression was not significantly correlated in the sample as a whole. However, in older women, known to have a twofold increased risk of depression compared to older males (Alexopoulos, 2005), this correlation was found to be statistically significant. This result points to the importance of taking sex into account in studies of older participants.

Older adults constitute the fastest growing demographic group in the industrialized world (Wetherell, 2010), with an increased risk for a neurodegenerative disorder. In an early stage, symptoms of a disorder of depression may be difficult to distinguish from depressive symptoms that may be a cardinal symptom of the MCI associated with dementia (Richard et al., 2013). Knowledge concerning the relation between depressive symptoms and cognitive aging will be important both when the primary problems are related to a disorder of depression and when related to an early stage of a neurodegenerative disorder (Alexopoulos, 2005; Areán et al., 2010; Wetherell, 2010).

Depression in old age is not as frequent as in younger adults, probably because older people tend to be better at regulating their emotional states (Mather, 2012). On the other hand, structural and functional changes in the aging brain may cause both cognitive decline and an increase in depressive symptoms (Anderton, 2002; Jung and Haier, 2007; Mather, 2012). With deterioration of the prefrontal cortex (PFC), there is a strong association with depressive symptoms in older-age. The PFC is also shown to be important to the cognitive ability tasks that comprise gF in this study (Jung and Haier, 2007), which might provide an explanation of the link between age and gF in the present study. Furthermore, cognitive speed measures, which the gF factor in this study relies heavily on, are shown to symptomatically decline in older adults with depression (Herrmann et al., 2007).

The impact of sex on symptoms of depression and the association between those symptoms and cognitive function need to be commented on. First of all, the sex-differences may partly be related to characteristics of the selected measures of cognitive function, defined within the concepts of gF and gC. Cattell (1963) hypothesized that gF would be a form of processing capacity, whereas gC would simply be a passive storage house for gained knowledge. Perhaps this conceptualization of intelligence needs to be redefined. Others have argued that the Vocabulary test is not a test for “intelligence as product,” as Deary et al. (2010) stated it, but rather reflects a current verbal intelligence ability (Johnson and Bouchard, 2005). Due to overall higher verbal abilities in females than males, the test is expected to favor females (Delis and Kramer, 2000; Lezak et al., 2004). Furthermore, depression is shown to have a negative impact on structural and functional measures related to verbal ability (Herrmann et al., 2007). It is thus not surprising that symptoms of depression would have a negative impact on verbal ability, creating this at first seemingly discrepant finding of gC being influenced by symptoms of depression in males. However, it is still surprising that we would find this effect in comparatively low levels of depressive symptoms, and not in females.

A more social explanation of the sex-differences revealed in the present study is based on results from studies showing that women tend to report more symptoms on mental health surveys than men (Tousignant et al., 1987). This may reflect that males are less depressed, but may also indicate that male participants tend to “under-report” symptoms of depression that should have caught medical attention (Piccinelli and Wilkinson, 2000; Alexopoulos, 2005), or that they express and interpret depressive symptoms different from most females (Rabbitt et al., 1995; Ng et al., 2009). The latter may be due to cultural and age-specific differences, with males more inclined socially to avoid expressing depressive symptoms, as this would be in conflict with norms regarding the male role and social interaction (Piccinelli and Wilkinson, 2000).

Historically, depression has been under-diagnosed in older adults (Alexopoulos, 2005). Herrmann et al. (2007) found in their systematic review and meta-analysis that late onset depression has a more severe effect on cognitive functioning than early onset depression. In middle- and older-age, cognitive as well as emotional functioning is of great importance, both in relation to work and daily activities, making it an imperative that this group receives adequate attention. Costs of subclinical depressive symptoms are expected to be large, not only at the individual level, but also at a societal level due to loss of productivity (Cuijpers et al., 2007). Recently, the term cognitive epidemiology has been suggested (Batty and Deary, 2004), as a means of incorporating measures of broad cognitive functioning (IQ) in epidemiological research. This line of research is of interest also in the traditions of cognitive reserve (Stern, 2002) and successful aging (Rowe and Kahn, 1997), where the emphasis is on finding factors related to retaining high cognitive functioning in old age. The present study, showing the importance of identifying even mild symptoms of depression in middle-aged and older men, fits well within this tradition.

### Limitations

The participants in the study represent a highly intelligent and well-educated self-selected sample. This may have confounding, and for the participants beneficial effects on cognitive abilities, indicating a large cognitive reserve (Stern, 2009). The sample is thus not representative of the whole population.

The aim was to study the effects of subthreshold depressive symptoms on normal aging in a sample screened for major disorders. The level of depressive symptoms in this study is not assessed using diagnostic tests or the criteria from the DSM or ICD, but is obtained from a dimensional self-report questionnaire (BDI). However, more subtle effects of depressive symptoms on cognitive function would be missed if we were to adhere to strict clinical cut-offs. The BDI has been used as an overall indicator of depressive symptoms; separate analyses including subscales of the BDI have not been performed, as our sample included healthy participants with no reason to assume a differentiated load on any such subscale. There is reason to assume that having a more representative sample, or a sample with marked cognitive deficits and/or other clinically significant disorders, the association between cognition and depressive symptoms would be even greater (Meeks et al., 2011).

### Implications

The key finding in the current study is that minimal symptoms of depression have a substantial negative correlation with gF in middle-aged and older males. Previous studies have found similar trends, indicating an increased susceptibility of depressive symptoms over a range of different domains for men, including social functioning and role impairment (Scott and Collings, 2010). Here we found that cognitive functioning is affected in men at low levels of depressive symptoms. Cognitive impairment has societal and economical implications, both as a risk for decreased work participation (Cuijpers et al., 2007), and as a risk factor for developing major depression (Meeks et al., 2011) and other morbidities (Bush et al., 2001). Minor and subthreshold symptoms of depression are more prevalent than full-scale syndromal major depression (Meeks et al., 2011), and are thus relevant both clinically and societally.

## Conclusion

Relatively minor symptoms of depression had a large effect on cognitive function. Interestingly, this held true only for males. This finding should be followed up by longitudinal analyses to investigate potential casual relationships between depressive symptoms and cognitive functioning. Our findings indicate that even minor symptoms of depression in middle-aged and older men should be taken seriously, as this may have an impact on their overall daily functioning. Reduced functioning in older adult males is costly, both at an individual and societal level.

## Conflict of Interest Statement

The authors declare that the research was conducted in the absence of any commercial or financial relationships that could be construed as a potential conflict of interest.
